# An In Vitro Model for *Candida albicans–Streptococcus gordonii* Biofilms on Titanium Surfaces

**DOI:** 10.3390/jof4020066

**Published:** 2018-06-04

**Authors:** Daniel Montelongo-Jauregui, Anand Srinivasan, Anand K. Ramasubramanian, Jose L. Lopez-Ribot

**Affiliations:** 1 Department of Biology, The University of Texas at San Antonio, San Antonio, TX 78249, USA; Daniel.Montelongo@utsa.edu; 2 South Texas Center for Emerging Infectious Diseases, The University of Texas at San Antonio, San Antonio, TX 78249, USA; 3 Research and Development, BioBridge Global, San Antonio, TX 78201, USA; anand.srinivasan@live.com; 4 Department of Biomedical, Chemical, and Materials Engineering, San Jose State University, San Jose, CA 95192, USA; anand.ramasubramanian@sjsu.edu

**Keywords:** titanium alloy, *Candida albicans*, *Streptococcus gordonii*, mixed biofilms, synthetic saliva

## Abstract

The oral cavity serves as a nutrient-rich haven for over 600 species of microorganisms. Although many are essential to maintaining the oral microbiota, some can cause oral infections such as caries, periodontitis, mucositis, and endodontic infections, and this is further exacerbated with dental implants. Most of these infections are mixed species in nature and associated with a biofilm mode of growth. Here, after optimization of different parameters including cell density, growth media, and incubation conditions, we have developed an in vitro model of *C. albicans–S. gordonii* mixed-species biofilms on titanium discs that is relevant to infections of peri-implant diseases. Our results indicate a synergistic effect for the development of biofilms when both microorganisms were seeded together, confirming the existence of beneficial, mutualistic cross-kingdom interactions for biofilm formation. The morphological and architectural features of these dual-species biofilms formed on titanium were determined using scanning electron microscopy (SEM) and confocal laser scanning microscopy (CLSM). Mixed biofilms formed on titanium discs showed a high level of resistance to combination therapy with antifungal and antibacterial drugs. This model can serve as a platform for further analyses of complex fungal/bacterial biofilms and can also be applied to screening of new drug candidates against mixed-species biofilms.

## 1. Introduction

Due to superior biocompatibility as well as mechanical and anticorrosive properties, titanium and its alloys have become the biomaterial of choice for a number of biomedical devices [1,2,3]. As a result, titanium-based implants are now frequently used in the healthcare industry [4]. More specifically in the field of dentistry, implant-supported dental restorations constitute a common treatment for the replacement of missing teeth, with titanium alloy Ti–6A1–4V representing the most commonly used material for bone-contacting dental implants [4]. However, the overall success of these dental implants is commonly and severely compromised by infection, including peri-implant mucositis and peri-implantitis [5,6,7]. These infections start with the adhesion and colonization of different pathogenic microorganisms (typically bacteria) on the implant surface, and subsequent biofilm formation [8]. Ultimately, these oral biofilms are complex microbial communities harboring a large number of species, including bacteria and fungi [9,10,11,12]. Among bacteria, several species have been described to play a role in the pathogenesis of periodontal and peri-implantar diseases [13,14]. Concerning fungi, *Candida* spp. (and, particularly, *C. albicans*) represent the most common fungal species in the oral cavity, and their presence has been reported in clinical studies assessing microbiota from healthy and failed implants [15,16,17], as well as having been associated with other implant infections such as denture stomatitis [18]. Multiple studies have attempted to elucidate the complexity of the oral microbiota associated with successful integration of dental implants and peri-implant soft tissues, which has been associated with the presence of Gram-positive cocci (including Streptococci), non-motile bacilli, and few Gram-negative cocci [19,20,21,22]. Conversely, the microbiota linked to dental implant failure has been reported to correlate with a higher prevalence of periodontal pathogens, including *Porphyromonas gingivalis*, *Prevotella intermedia*, and Gram-negative cocci, together with *Candida* spp. [5,23,24,25,26,27]. 

Regarding titanium surfaces, the adhesion and subsequent colonization of dental implants by different bacterial species has been widely reported in the scientific literature [28]. Somewhat surprisingly, the same is not true in the case of *Candida* spp., with very limited information about the adhesive and biofilm-forming abilities of these opportunistic fungi to this commonly used biomaterial, with rare exceptions [29,30,31,32,33]. Furthermore, these infections are often polymicrobial in nature, including both bacteria and fungi, but no detailed study exists on the formation of mixed bacterial/fungal biofilms on titanium surfaces. In a previous study we reported on the morphological and architectural characteristics of mixed biofilms formed by *C. albicans* together with the oral bacterium *Streptococcus gordonii*, an early colonizer of oral surfaces, for which we used mostly an in vitro model utilizing standard 96-well polystyrene microtiter plates [34]. Here, expanding upon our earlier observations, we have developed an in vitro model that more closely resembles mixed *C. albicans*/*S. gordonii* oral biofilms formed on titanium implants. We have characterized the structure of the resulting dual-species biofilms, and demonstrated the synergistic interactions between these two microorganisms with regards to their ability to form biofilms on titanium surfaces and their corresponding antimicrobial resistance.

## 2. Materials and Methods

### 2.1. Microorganisms and Culture Conditions

We used *C. albicans* SC5314 strain and *S. gordonii* Challis DL1.1. *S. gordonii* was incubated anaerobically in a CO_2_ incubator on Tryptone Soy Agar (TSA) plates with 5% sheep blood from Remel (San Diego, CA, USA). Suspension cultures of *S. gordonii* were grown without shaking inside a 5% CO_2_ incubator for 16 h at 37 °C in 20 mL of Todd Hewitt Broth + 0.02% *w*/*v* Yeast Extract (THB + 0.02% YE). After 16 h incubation, 100 µL from the liquid culture was obtained, transferred to 10 mL of fresh THB + 0.02% YE media, and shaken in a MaxQ™ 4000 benchtop orbital shaker (Thermo Fisher Scientific, Waltham, MA, USA) at 180 rpm for 3 h at 37 °C. Cells were recovered by centrifugation; the pellet was resuspended and washed in sterile PBS. *C. albicans* was routinely propagated aerobically on Yeast Peptone Dextrose (YPD) agar plates at 30 °C. Suspension cultures were grown overnight in 20 mL of YPD broth at 28 °C placed in a New Brunswick Scientific (Edison, NJ, USA) gyratory water bath shaker at 150–180 rpm. From these cultures, fungal cells were recovered by centrifugation; the resulting pellet was resuspended and washed in sterile PBS. Finally, microbial cells were resuspended in either RPMI 1640 medium, THB + 0.02% YE medium, a 1:1 *v*/*v* mixture of RPMI 1640/THB + 0.02% YE media (referred to as 1:1 media from now on), or basal medium mucin (BMM) synthetic saliva. These suspensions were standardized to 1.0 × 10^6^ yeast cells/mL after counting using a hemocytometer and to 1.0 × 10^7^ bacterial cells/mL, calculated by measuring OD_600_ with a spectrophotometer.

### 2.2. Composition and Preparation of Basal Medium Mucin (BMM) Synthetic Saliva Medium

BMM synthetic saliva was prepared as described before [34] with slight modifications [35]. The composition per liter is as follows: 2.5 g partially purified pig gastric mucin, 5 g protease peptone, 5 g yeast extract, 2.5 mg haemin, 1 mg menadione, 33.5 mmol KCl, 1 mmol urea, and 1 mmol arginine.

### 2.3. Antimicrobial Drugs

The antibiotics used in this study included fluconazole (Hospira, Lake Forest, IL, USA), amphotericin B (Gibco Life Technologies, Grand Island, NY, USA), caspofungin (Merck & Co., Inc., Whitehouse Station, NJ, USA), and clindamycin (RPI Corp., Prospect, IL, USA).

### 2.4. Titanium Preparation, Cleaning, and Sterilization

Titanium Ti–6Al–4V was obtained in 12 × 12 inch sheets with a thickness of 0.016 inches from McMaster-Carr (Atlanta, GA, USA). The titanium sheets were cut to obtain ~900 circular discs per sheet with 7 mm diameter; this size was used to approximate the surface area of each well in a flat bottom 96-well microtiter plate.

For sterilization of titanium discs, we used the passivation method following the standard American Association for the International Association for Testing Materials (ASTM, West Conshohocken, PA, USA) F86 with slight modifications. In short, titanium alloy discs were placed in a 50 mL beaker submerged in 70% acetone and sonicated using a Branson 2510 Ultrasonic Cleaner (Branson Ultrasonics, Danbury, CT, USA) for 10 min, followed by 10 min sonication in ethanol and 10 min sonication in deionized water. Following sonication, titanium discs were placed in a 3:7 (*v*/*v*) nitric acid–deionized water solution for 30 min at room temperature. Discs were rinsed thoroughly with deionized water and sonicated 3 additional times for 15 min in deionized water. Finally, discs were dried using nitrogen gas and were individually placed in each well of a 48-well microtiter plate.

Once the plate was fully loaded with one disc in each well, the plate was sealed and stored at room temperature. To ensure discs were sterile after storage, plates were placed under UV light for 30 min on the day of experiment prior to addition of cells.

### 2.5. Biofilm Formation on Titanium Discs

Bacterial and fungal cell seeding was done by adding 300 µL per well (sufficient to entirely cover the titanium surface) of the prepared single- or mixed-cell suspension in 1:1 media or BMM synthetic saliva for comparison. The final cell concentrations were *C. albicans* at 1 × 10^6^ cell/mL and *S. gordonii* at 1 × 10^7^ cell/mL. After seeding, the 48-well plates were incubated in a 5% CO_2_ incubator for 24 h at 37 °C. Presto Blue™ Cell Viability Reagent (Invitrogen™, Carlsbad, CA, USA) was used for estimation of biofilm formation on titanium discs as described before by our group [36]. Briefly, after incubation, the supernatant was removed and samples were washed twice with 300 µL PBS. The PBS was aspirated and the viability of cells within the biofilms was estimated by adding 300 µL of 1:10 *v*/*v* Presto Blue™ reagent in 1:1 media and incubating for 1 h inside a 5% CO_2_ incubator at 37 °C. Finally, 240 µL from each well was transferred into a new 48-well plate for fluorescent readings. The microtiter plate reader (BioTek^®^ Synergy™ HT, Winoosky, VT, USA) was set to measure fluorescence at 530/25 nm excitation and 590/35 emission.

### 2.6. Kinetic Studies on the Formation of Mixed Biofilms on Titanium

After seeding fungal and bacterial cells on titanium discs (as described above), discs containing microorganisms were collected every 4 h for a period of 24 h, in triplicate. Plates were washed twice with PBS. Cell viability was measured by adding 300 µL of 1:10 *v*/*v* Presto Blue™ as described above. In addition, biofilm formation at different time points was monitored by fluorescence microscopy. Briefly, biofilms formed on titanium were stained in the dark in the following order: at 37 °C for 30 min with 25 µg/mL concavalin A–Alexa Fluor^®^ 488 conjugate (Molecular Probes, Eugene, OR, USA) to label the fungal cell wall; at 37 °C for 20 min with 5 µg/mL wheat germ agglutinin, Texas Red™-X Conjugate (WGA, Molecular Probes) to label the bacterial cell wall (although a caveat here is that WGA can also bind to chitin in the fungal cell wall); and at 37 °C for 10 min with 300 nM 4′,6-diamidino-2-phenylindole, dihydrochloride (DAPI, Molecular Probes) to label nucleic acids. These samples were visualized using a 63× objective lens in a Leica DMR fluorescent microscope (Leica Microsystems, Buffalo Grove, IL, USA).

### 2.7. Scanning Electron Microscopy

Titanium discs with a biofilm on their surface were fixed with glutaraldehyde (2.5% *w*/*v*)–0.1 M sodium calcodylate buffer at pH 7.4 (Electron Microscopy Sciences, Hatfield, PA, USA) for 2 h at 37 °C. After fixation, the glutaraldehyde solution was removed and osmium tetroxide solution (1% *w*/*v*)–0.1 M sodium cacodylate buffer at pH 7.4 was added to the samples for 2 h at room temperature (Electron Microscopy Sciences). The titanium discs with biofilms were then rinsed with water and dehydrated in a graded series of ethanol concentrations. Samples were coated with a 60:40 gold–palladium alloy using a sputter coater and visualized using a JEOL JSM-6610 Scanning Electron Microscope (JEOL USA, Inc., Peabody, MA, USA).

### 2.8. Confocal Laser Scanning Microscopy

Biofilms on titanium were stained in a sequential order starting with 25 μg/mL Concanavalin A–Alexa Fluor^®^ 488 conjugate (excitation/emission; 495/519) (Molecular Probes, Eugene, OR, USA) for 30 min at 37 °C, followed by 1 x FilmTracer™ SYPRO^®^ Ruby Biofilm Matrix Stain (Molecular Probes) for 30 min at room temperature, and finally with 300 nM 4′,6-diamidino-2-phenylindole, dihydrochloride (DAPI) (excitation/emission; 358/461) (Molecular Probes, Eugene, OR, USA) for 10 min at 37 °C. Fluorescent stains were removed and discs were rinsed by immersion (two times) in sterile PBS. Biofilms were visualized with an Achroplan 63x-oil objective lens using a Zeiss LSM 510 upright confocal microscope (Carl Zeiss, Thornwood, NY, USA). Pictures were analyzed and processed using AutoQuant X2 (Media Cybernetics, Rockville, MD, USA).

### 2.9. Antimicrobial Susceptibility Testing of Dual-Species Biofilms Formed on Titanium

Specific drug dilutions were prepared from stock solutions in either 1:1 media or BMM synthetic saliva and were added to preformed mixed biofilms formed on the titanium discs for 24 h. In addition, no-drug control discs and dead cell control discs treated with 10% Triton^®^ X-100 detergent (Fisher Bioreagents^®^, Fair Lawn, NJ, USA) were used in each plate. Plates were incubated in the presence of antimicrobials for an additional 24 h. Finally, discs were rinsed in sterile PBS and processed using the Presto Blue™ assay as described above.

### 2.10. Statistics

Prism software (GraphPad, La Jolla, CA, USA) was used to statistically analyze the data using one-way ANOVA and Dunnett’s test for multiple comparisons. Biofilm formation assays of single- and mixed-species biofilm were performed in two independent plates, with each condition tested in triplicate. Statistically significant differences were considered if *p* < 0.05. Antimicrobial susceptibility testing assays were also performed in two independent plates, with three equivalent wells per condition. Using the Presto Blue™-readings, normalization of data was done with respect to the average of 6 no-drug samples considered as 100%, and to the average of 6 dead cell control samples treated with 10% Triton X-100 considered as 0%.

## 3. Results

### 3.1. Development of C. albicans and S. gordonii Single- and Dual-Species Biofilms on Titanium Discs

We developed an in vitro model of single- and mixed-species biofilms of *C. albicans* and *S. gordonii* on titanium alloy Ti–6Al–4V. Briefly, titanium sheets were cut into discs of the appropriate size. After preparation (see Section 2.4), individual titanium discs were inserted into the wells of 48-well microtiter plates. Studies were performed in which *C. albicans* and *S. gordonii* were seeded alone or in combination, and using different media including RPMI 1640, THB + 0.02% YE, a combination of the two at a 1:1 ratio, and BMM synthetic saliva, that more closely resembles physiological conditions encountered by microorganisms within the oral cavity. After seeding the cells, the plates were then incubated in a 5% CO_2_ incubator for 24 h at 37 °C to allow for biofilm formation. Finally, the titanium discs were removed and transferred to a new plate for estimation of biofilm formation using the Presto Blue™ metabolic readings.

**Figure 1:**
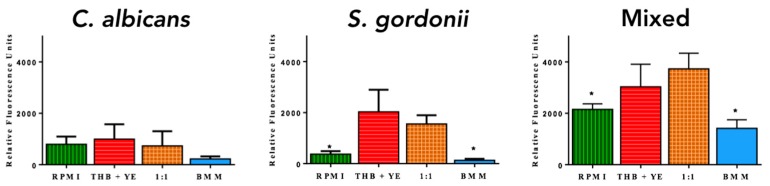
Metabolic activity after 24 h incubation of *C. albicans* and *S. gordonii* single- and dual-species biofilms formed on titanium. Biofilms were grown in RPMI 1640, THB + 0.02% YE, 1:1 *v*/*v* RPMI/THB + YE and BMM synthetic saliva in a 48-well plate containing titanium discs for 24 h. After incubation, discs were placed inside a new, sterile 48-well plate and viability was measured by Presto Blue^TM^ fluorescence; *n* = 3. Error bars represent standard deviations. * indicates statistically significant differences.

*C. albicans* and *S. gordonii* monospecies biofilms were most proliferative in THB + 0.02% YE (Figure 1) as compared to the other media used. This result was expected for *S. gordonii*, but not anticipated for *C. albicans* biofilms because RPMI has been the medium of choice for the formation of fungal biofilms [37]. However, dual-species biofilms grew best in 1:1 media (Figure 1). Irrespective of the media used, the two microorganisms together formed more robust biofilms compared to the single-species biofilms, clearly pointing to the synergistic interactions of these two microorganisms in dual-species biofilms. For example, although single-species biofilms were least proliferative in BMM synthetic saliva, when both bacterial and fungal cells were seeded together in this medium there was robust biofilm formation on the titanium discs as indicated by much higher fluorometric readings. Overall, these results demonstrate the ability of both microorganisms to grow on the dental implant material under all conditions tested.

We also performed a series of biofilm kinetic studies using fluorescent microscopy over a period of 24 h to monitor the formation of mixed-species biofilms of *C. albicans* and *S. gordonii* on titanium discs, using both 1:1 and BMM media. For these studies we used Concanavalin A to stain the fungal wall, Wheat Germ Agglutinin to stain the bacterium (although we note that it also binds chitin on the fungal cell wall), and DAPI for nucleic acid. As shown in Figures S1 and S2, *C. albicans* germ tube formation was observed as early as the 4 h time point in both 1:1 media and BMM saliva, with extensive filamentation observed at 8–12 h in the case of BMM synthetic saliva and at about 16 h in the case of 1:1 media. We also observed increased accumulation of *S. gordonii* with biofilm maturation, always in close association with *C. albicans* filaments.

### 3.2. Structural Characteristics of C. albicans and S. gordonii Single- and Dual-Species Biofilms Formed on Titanium Discs 

**Figure 2:**
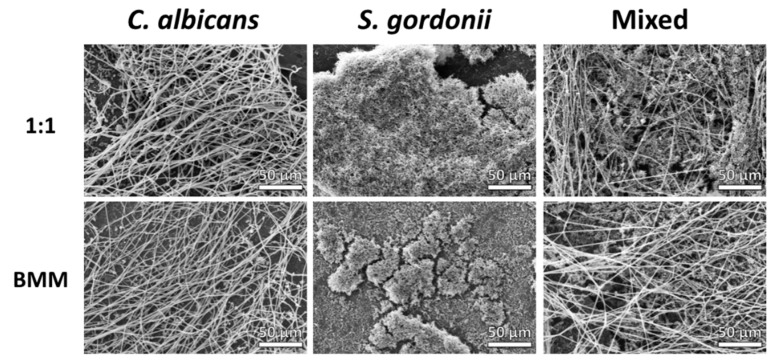
SEM micrographs of *C. albicans* and *S. gordonii* single- and dual-species biofilms grown in 1:1 *v*/*v* RPMI/THB + 0.02% YE media (upper panel) and BMM synthetic saliva (lower panel) formed on titanium discs. Magnification ×500.

We performed scanning electron microscopy (SEM) in order to directly visualize the single- and dual-species biofilms formed on titanium in 1:1 and BMM media (Figure 2). Extensive filamentation was observed in *C. albicans* biofilms formed using both media, although we observed slightly higher cell density in 1:1 media as compared to in BMM synthetic saliva. Similarly, *S. gordonii* formed dense biofilms covering most of the titanium surface under both conditions used. These biofilms appeared rather homogeneous when grown in 1:1 media; however, increased clustering of bacterial cells and overall structural heterogeneity was observed in *S. gordonii* single-species biofilms formed in BMM synthetic saliva. SEM observations of dual-species *C. albicans*/*S. gordonii* biofilms showed extensive interactions between both microorganisms, irrespective of media conditions used, with extensive filamentation in *C. albicans* cells and close interactions between bacterial aggregates and hyphal elements (see also Figure S3 showing higher-magnification microphotographs).

**Figure 3:**
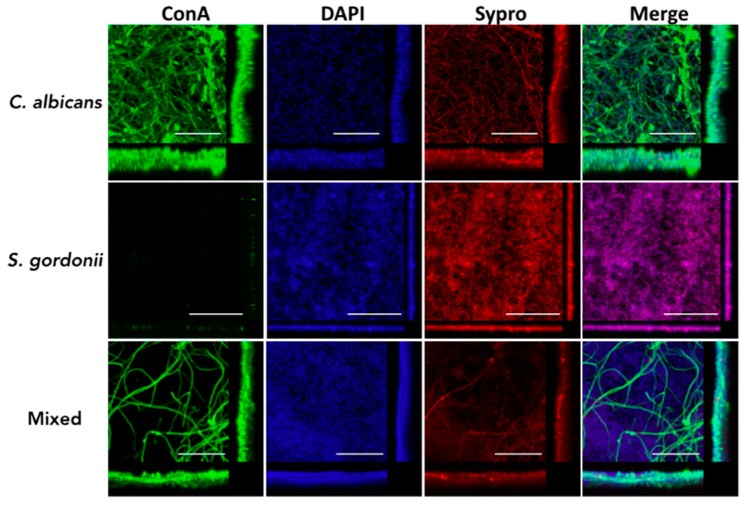
Characterization of *C. albicans* and *S. gordonii* single- and dual-species biofilms formed on titanium using confocal laser scanning microscopy (CLSM) and 3D reconstruction software. Biofilms were grown for 24 h in 1:1 *v*/*v* RPMI/THB + YE in a 48-well plate containing titanium discs, and stained with Concanavalin A-Alexa Fluor^®^ 488 conjugate, 1 x FilmTracer™ SYPRO^®^ Ruby Biofilm Matrix Stain, and DAPI. Scale bar corresponds to 50 µm. Included in the figure are the *xy*, *xz*, and *yz* views of the resulting biofilm.

**Figure 4:**
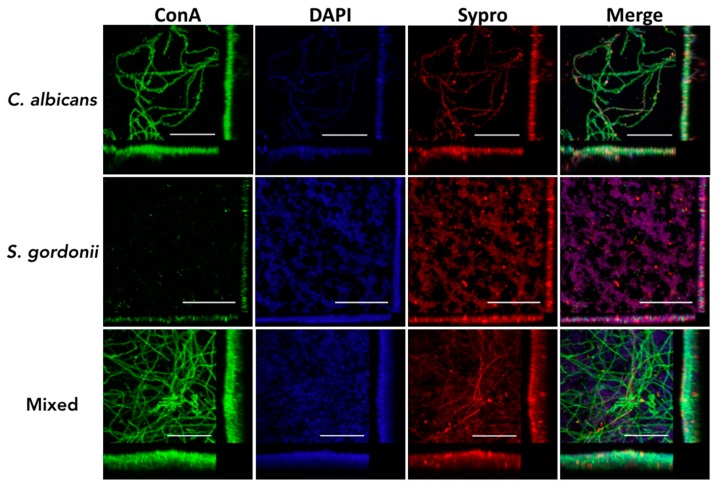
Characterization of *C. albicans* and *S. gordonii* single- and dual-species biofilms formed on titanium using CSLM and 3D reconstruction software. Biofilms were grown for 24 h in BMM synthetic saliva in a 48-well plate containing titanium discs, and stained with Concanavalin A-Alexa Fluor^®^ 488 conjugate, 1 x FilmTracer™ SYPRO^®^ Ruby Biofilm Matrix Stain, and DAPI. Scale bar corresponds to 50 µm. Included in the figure are the *xy*, *xz*, and *yz* views of the resulting biofilm.

We also used confocal laser scanning microscopy (CLSM) to further examine the main architectural features and three-dimensional characteristics of *C. albicans* and *S. gordonii* single- and dual-species biofilms formed on the titanium discs, using both 1:1 media and BMM synthetic saliva. Confirming results from metabolic readouts obtained using Presto Blue, both *C. albicans* and *S. gordonii* were able to form robust single-species biofilms on the titanium alloy surface using 1:1 media (Figure 3); however, this was not the case in BMM synthetic saliva (Figure 4). For example, although filamentation was observed in *C. albicans* single-species biofilms formed in BMM synthetic saliva, the resulting biofilm on the titanium surface showed less confluency and overall thickness when compared with an equivalent biofilm formed in 1:1 media. Likewise, *S. gordonii* single-species biofilms formed in BMM synthetic saliva consisted of relatively sparse microcolonies, as opposed to a much more confluent and dense biofilm covering the entire the titanium surface when formed using 1:1 media (compare Figure 3 and Figure 4). The interactions between the two microorganisms resulted in a much higher degree of complexity and heterogeneity of the mixed fungal/bacterial biofilms (Figure 3 and Figure 4). Also confirming the results from Presto Blue metabolic readings, the synergism between the two species was particularly noticeable in the case of dual-species biofilms formed in BMM synthetic saliva, with strong biofilm formation on the titanium surface when the two organisms were seeded together as compared with the relatively poor biofilms formed by each species alone (Figure 4).

### 3.3. Dual-Species Biofilms of C. albicans and S. gordonii Formed on Titanium Display High Levels of Resistance to Antimicrobial Therapy

**Figure 5:**
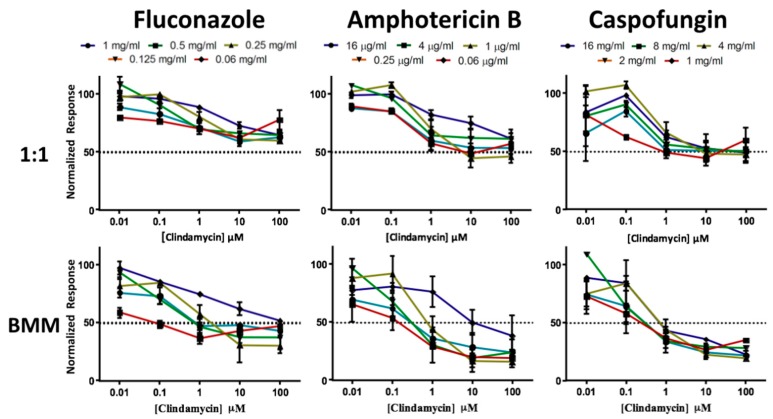
Antimicrobial susceptibility patterns of preformed dual-species *C. albicans/S. gordonii* biofilms formed in 1:1 *v*/*v* RPMI/THB + 0.02% YE media (1:1, upper panels) or BMM synthetic saliva (BMM, lower panels) using an antibacterial/antifungal combination therapy.

One of the most important consequences of biofilm formation is the increased resistance to antimicrobial treatment, and this is particularly problematic in the case of polymicrobial biofilms. Thus, we examined the susceptibility properties of *C. albicans*/*S. gordonii* dual-species biofilms formed on a titanium surface, grown in both 1:1 media and BMM synthetic saliva, against combinations of antifungal and antibacterial drugs. For this set of experiments, we allowed these mixed biofilms to form on titanium discs for 24 h as described above. Once formed, biofilms were washed and exposed to antimicrobials for an additional 24 h. We tested three common antifungals, including fluconazole, amphotericin B, and caspofungin as representatives of each of the three major classes of antifungal drugs (azoles, polyenes, and echinocandins) normally used to treat *Candida* infections. As an antibacterial, we used clindamycin, a widely used antibiotic for treatment against Gram-positive bacteria. As shown in Figure 5, mixed biofilms formed on titanium were highly resistant to combinations of these antimicrobials. This was particularly true in 1:1 media, where even high concentrations of both antifungal and antibacterial drugs were not able to achieve a >50% reduction in metabolic readings. Although mixed biofilms formed using BMM synthetic saliva showed a slightly increased susceptibility to antimicrobial treatment, we note that >50% inhibition was only achieved at relatively high concentrations of both the antifungal and antibacterial agents. Overall, these results demonstrate that mixed fungal/bacterial biofilms formed on titanium are highly recalcitrant to antimicrobial treatment, thereby complicating treatment of these implant infections.

## 4. Discussion

The use of dental implants has increased in recent years, and according to the American Academy of Implant Dentistry (AAID), it is estimated that the U.S. and European market for dental implants is expected to reach $4.2 billion by 2022 [38,39,40,41]. Titanium—more specifically, its alloy Ti–6A1–4V—represents the most commonly used material for dental implants [4]. Infection constitutes one of the main causes of failure of dental implants, since they are exposed to a complex oral microbiota and these biomaterials can support microbial adherence and colonization during or after surgical implantation [5,6,7,8]. In particular, microbial biofilms developed on dental implants play a major role in the pathogenesis of these infections [8]. Therefore, there is a need to better understand microbial biofilm formation on implant materials.

Previous reports have shown that normal oral commensals such as *S. gordonii* can stimulate virulence of *C. albicans* by increasing its adherence and inducing filamentation in oral tissues [42,43,44,45,46,47]. Here, we have extended our previous studies on *C. albicans*/*S. gordonii* mixed fungal/bacterial biofilms [36], and examined the ability of these two common microorganisms of the oral cavity to form single- and dual-species biofilms on titanium material, thus more closely mimicking conditions associated with dental implant infections. This is important since, to date, there has been very limited data on the ability of *Candida* spp. to form biofilms on titanium surfaces [29,30,31,32,33,48], and even less information regarding mixed fungal/bacterial biofilms. In the process, we have developed a novel model in which titanium discs are placed within wells of microtiter plates, allowing for the formation of multiple equivalent biofilms on the titanium surface in a highly reproducible manner.

Not surprisingly, our overall results point to the ability of both *C. albicans* and *S. gordonii* to readily form monospecies biofilms on titanium surfaces. Also, as reported in our previous study using polystyrene plates [36], we observed a strong synergy between these two organisms in their ability to form dual-species biofilms on titanium surfaces, which was particularly noticeable when using the more physiological BMM synthetic saliva medium. Using advanced microscopy techniques, we further characterized the morphological and architectural features of these dual-species biofilms formed on titanium, and demonstrated the intimate interactions between fungal elements and bacterial cells in the resulting fungal/bacterial biofilms. Moreover, these dual-species biofilms are intrinsically resistant to antimicrobial treatment, even when high concentrations of antifungals and antibacterial are used together, pointing to the difficulties in the therapy of this type of implant infection.

In summary, we have developed an in vitro model that more closely resembles mixed *C. albicans*/*S. gordonii* oral biofilms on titanium implants, characterized their structural characteristics, and demonstrated the synergistic interactions between these two microorganisms in regards to biofilm formation and antimicrobial resistance. Results point to the need for new effective approaches to combat the threat of polymicrobial implant infections. This model also serves as a platform for further analyses of complex fungal/bacterial biofilms and can be applied to the screening of new drug candidates against mixed-species biofilms.

## Supplementary Materials

The following are available online at http://www.mdpi.com/2309-608X/4/2/66/s1.
