# Supplementary material for: An In Vitro Model for Candida albicans–Streptococcus gordonii Biofilms on Titanium Surfaces
Source: J Fungi (Basel). 2018 Jun 4;4(2):66. doi: 10.3390/jof4020066 (PMC6023327; doi:10.3390/jof4020066)
Supplement: Supplementary file 1 [file jof-04-00066-s001.pdf]

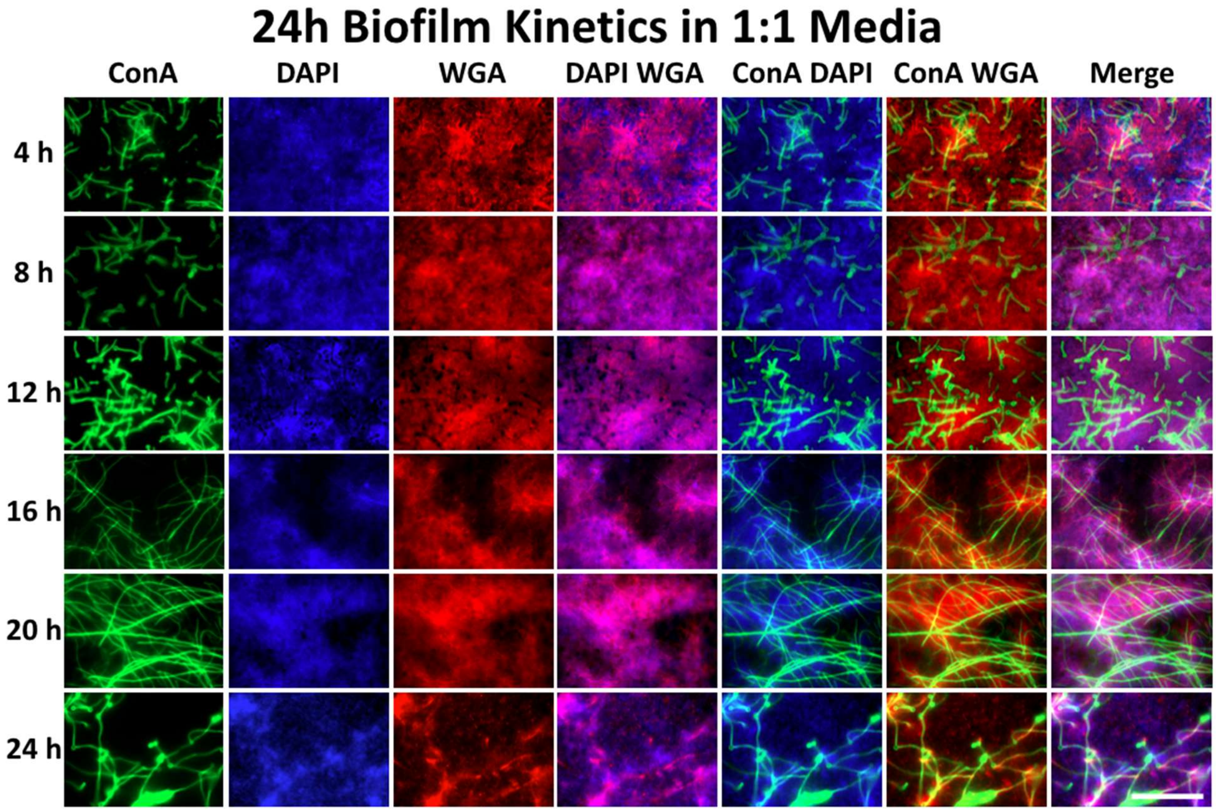

Figure S1. Kinetic study for the characterization of mixed species biofilms of *C. albicans* and *S. gordonii* using fluorescence microscopy. Mixed biofilms grown in 1:1 media were stained using Concavalin A—Alexa Fluor 488 fungal cell wall stain (green), DAPI nucleic acid stain (blue) and Wheat Germ Agglutinin (red). Scale bar is 100 μm for all panels.

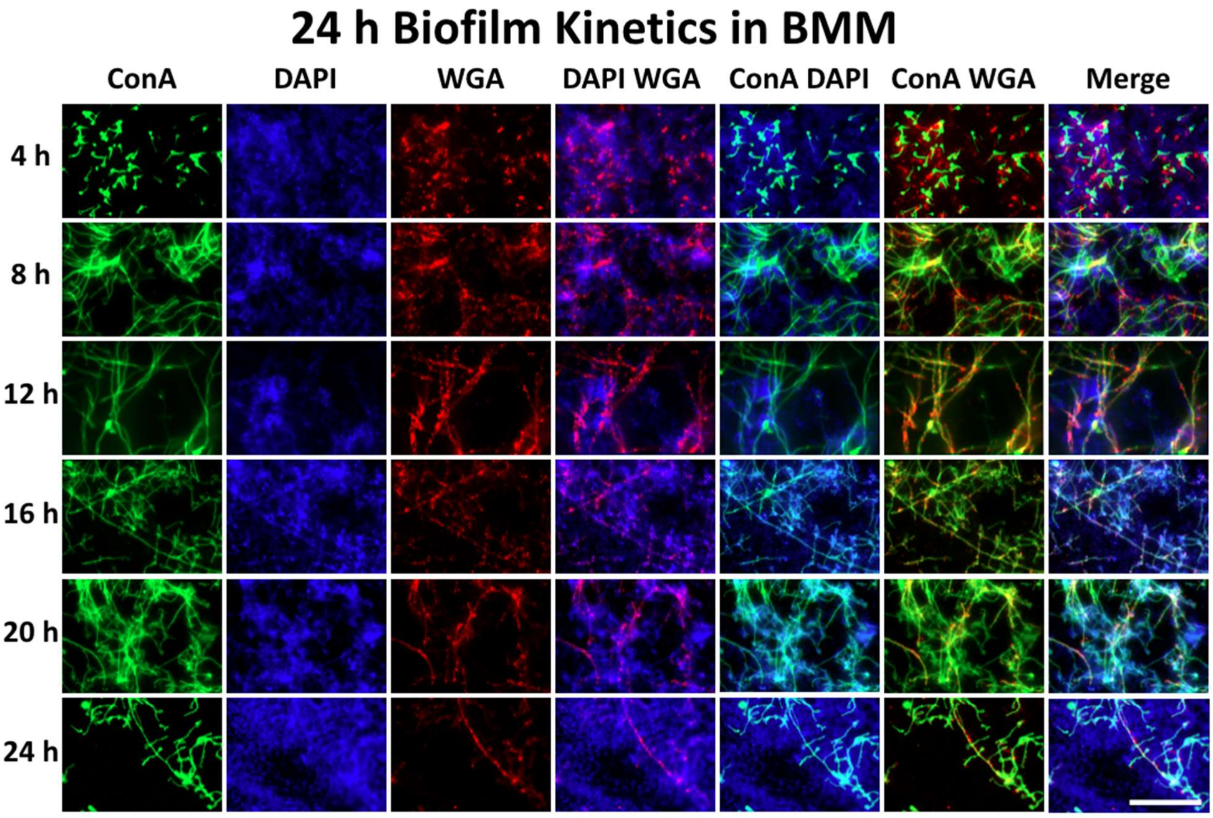

Figure S2. Kinetic study for the characterization of mixed species biofilms of *C. albicans* and *S. gordonii* using fluorescence microscopy. Mixed biofilms grown in BMM synthetic saliva were stained using Concavalin A—Alexa Fluor 488 fungal cell wall stain (green), DAPI nucleic acid stain (blue) and Wheat Germ Agglutinin (red). Scale bar is 100 μm for all panels.

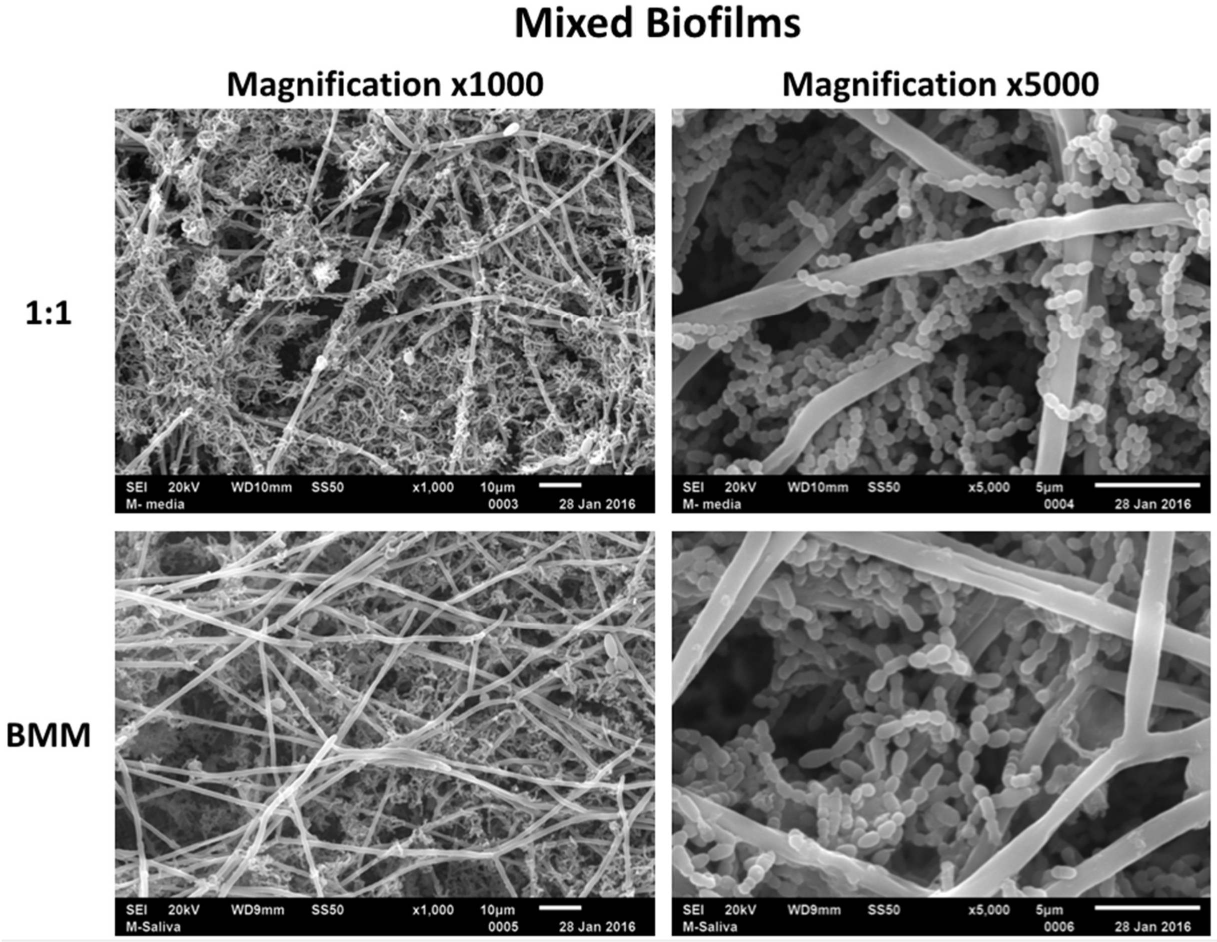

Figure S3. SEM micrographs of *C. albicans and S. gordonii* dual-species biofilms grown in 1:1 *v/v* RPMI/THB + 0.02% YE media (upper panel) and BMM synthetic saliva (lower panel) formed on titanium discs. Magnification ×1000 and ×5000.
